# Transcription factor network analysis based on single cell RNA-seq identifies that Trichostatin-a reverses docetaxel resistance in prostate Cancer

**DOI:** 10.1186/s12885-021-09048-0

**Published:** 2021-12-08

**Authors:** Patricia M. Schnepp, Aqila Ahmed, June Escara-Wilke, Jinlu Dai, Greg Shelley, Jill Keller, Atsushi Mizokami, Evan T. Keller

**Affiliations:** 1 Department of Urology, University of Michigan Medical School, NCRC B14 RM116, Ann Arbor, MI 48109 USA; 2 Unit for Laboratory Animal Medicine, University of Michigan, NCRC B14 RM116, Ann Arbor, MI 48109 USA; 3 Department of Urology, Kanazawa University, Kanazawa, Japan; 4 Biointerfaces Institute, University of Michigan, NCRC B14 RM116, Ann Arbor, MI 48109 USA; 5 Single Cell Spatial Analysis Program, University of Michigan, NCRC B14 RM116, Ann Arbor, MI 48109 USA

**Keywords:** Docetaxel resistance prostate cancer, Trichostatin a, Single cell RNA sequencing, PANDA method, Transcription factor network analysis

## Abstract

**Background:**

Overcoming drug resistance is critical for increasing the survival rate of prostate cancer (PCa). Docetaxel is the first cytotoxic chemotherapeutical approved for treatment of PCa. However, 99% of PCa patients will develop resistance to docetaxel within 3 years. Understanding how resistance arises is important to increasing PCa survival.

**Methods:**

In this study, we modeled docetaxel resistance using two PCa cell lines: DU145 and PC3. Using the Passing Attributes between Networks for Data Assimilation (PANDA) method to model transcription factor (TF) activity networks in both sensitive and resistant variants of the two cell lines. We identified edges and nodes shared by both PCa cell lines that composed a shared TF network that modeled changes which occur during acquisition of docetaxel resistance in PCa. We subjected the shared TF network to connectivity map analysis (CMAP) to identify potential drugs that could disrupt the resistant networks. We validated the candidate drug in combination with docetaxel to treat docetaxel-resistant PCa in both in vitro and in vivo models.

**Results:**

In the final shared TF network, 10 TF nodes were identified as the main nodes for the development of docetaxel resistance. CMAP analysis of the shared TF network identified trichostatin A (TSA) as a candidate adjuvant to reverse docetaxel resistance. In cell lines, the addition of TSA to docetaxel enhanced cytotoxicity of docetaxel resistant PCa cells with an associated reduction of the IC50 of docetaxel on the resistant cells. In the PCa mouse model, combination of TSA and docetaxel reduced tumor growth and final weight greater than either drug alone or vehicle.

**Conclusions:**

We identified a shared TF activity network that drives docetaxel resistance in PCa. We also demonstrated a novel combination therapy to overcome this resistance. This study highlights the usage of novel application of single cell RNA-sequencing and subsequent network analyses that can reveal novel insights which have the potential to improve clinical outcomes.

**Supplementary Information:**

The online version contains supplementary material available at 10.1186/s12885-021-09048-0.

## Background

Prostate cancer (PCa) is the second leading cause of cancer-related deaths in men in the United States [1]. While the first line of treatment for advanced PCa is androgen deprivation therapy, the majority of patients develop castrate-resistant PCa (CRPC) [2] which leads to use of chemotherapy. Docetaxel, a taxane, was one of the first cytotoxic therapies approved for CRPC in the United States [3]. It operates through the stabilization of microtubules and inhibition of Bcl-2 expression [4–6]. However, the survival benefits of docetaxel are limited with resistance developing in nearly 99% of patients within 3 years [7]. Understanding how this resistance arises is critical to identify strategies to overcome resistance and increase the survival of PCa patients.

While previous studies to delineate the mechanisms of docetaxel resistance in PCa have identified putative targets, these studies focused on a small number of gene expression changes that occur during drug resistance [8, 9]. In a previous study, we used single cell RNA-sequencing of docetaxel-resistant PCa cells to identify putative candidates of docetaxel resistance [10]. However, a limitation of that study was the lack of integrating the data with gene pathways and transcriptional activators in a more holistic fashion. An integrative and systems-level approach that, in addition to transcription expression, incorporates protein interactions and transcriptional activation may help to better understand the progression towards drug resistance and identify combination therapies to overcome resistance. The ability of such multifaceted integrated approaches that include information from multiple data sources to unveil important biological insights have become apparent in recent years [11–14]. In the current study, we adapted an integrative network inference method, Passing Attributes between Networks for Data Assimilation (PANDA), to model the transcription factor (TF) regulatory network in docetaxel sensitive and resistant PCa cell lines [15]. PANDA develops a regulatory model by iteratively integrating the information from TF-TF protein interaction, gene expression profile and gene co-regulation, and TF-binding motif data. This method has been previously successfully adapted to study ovarian cancer [16] and breast cancer [17] through analysis of bulk samples. In the current study, we applied PANDA for the first time to single cell transcriptomes. Single cell RNA sequencing (scRNA-seq) uncovers the variability and heterogeneity of individual cells in a population that cannot be appreciated using traditional bulk sequencing. This allows us to identify new information only observed through sequencing individual cells and provides a novel application of PANDA method to identify active networks in the development of docetaxel resistance.

In this study, we applied PANDA to characterize the TF regulatory network underlying development of docetaxel resistance in docetaxel sensitive and resistant variants of the PC-3 and Du145 PCa cell lines. We conducted scRNA-seq on the sensitive and resistant variations of both cell lines. We identified shared network nodes and edges between the two cell lines. We also identified the TFs driving resistance and validated their importance in maintaining drug resistance. Furthermore, we subjected the networks to connectivity map analysis (CMAP) to identify candidate therapeutics to reverse docetaxel resistance in PCa. Based on the CMAP, we identified trichostatin A as a candidate therapy and then demonstrated that TSA, in combination with docetaxel successfully decreased tumor growth in both in vitro and in vivo PCa models. This work provides valuable insight into a novel strategy using scRNA-seq to identify mechanisms of docetaxel resistance as well as candidate therapies to reverse drug resistance.

## Materials and methods

### Cell lines and reagents

DU145 (cat no. HTB-81) and PC3 (cat no. CRL-1435) were purchased from ATCC (Virginia, USA). The docetaxel resistant strains were created as previously described [9]. All cells were cultured in RPMI 1640 (Invitrogen Co., Carlsbad, CA) supplemented with 10% fetal bovine serum (FBS) and 1% penicillin-streptomycin (Life Technologies, Inc.). Resistance was maintained in the cells using growth media supplemented with 10 nM of docetaxel while sensitive cells were maintained with the addition of DMSO to a final level of 0.1% in the growth media (Cell Signaling Technology). Cell identification is confirmed annually using PCR for short tandem repeats.

### Gene expression quantification

The single cell samples were previously sequenced and published by our group [10]. In brief, for 1 week, cells were transferred to docetaxel free media. Cells were trypsinized in 0.05% Trypsin EDTA for 5–10 min at 37 °C and washed with media. For single cell sequencing, the cell suspension was loaded into in the Fluidigm C1™ machine and processed into single cell cDNA libraries according to manufacturer protocol (PN 101–4981). Briefly, full length mRNA-seq libraries were generated from single cells captured using the Fluidigm C1™ Single Cell mRNA Seq IFC, 10-17 μm (PN 100–5760) and Fluidigm C1™ Single-Cell Reagent Kit for mRNA Seq (PN 100–6201). Each chip was visually inspected to identify which wells contained cells. Wells containing one cell were included in library preparation ad sequencing. The capture rate was between 78 and 96% across all chips used in this study. Full length cDNA was converted into sequence ready libraries using SMART-seq v4 Ultra Low Input RNA kit for Fluidigm C1™ System (Takara Bio, Mountain View, CA, Cat 635,025) and SeqAmp™ DNA Polymerase (Takara Bio, Cat 638,504). Library preparation was completed using Nextera XT DNA library prep kit (Illumina, San Diego, CA, Cat. FC-131-1096) and Nextera XT DNA Library Prep Index Kit (Illumina, Cat FC-131-1002). Samples following PCR reactions as called for in each kit’s manufacturer’s protocol was purified using Agencourt AMPure XP (Beckman Coulter, Brea, CA, Item No A63880). Samples were sequenced on Illumina HiSeq-2500 Rapid for DU145 cell line variants and Illumina HiSeq-4000 with single end option for PC3 cell line variants. Reads that were below the minimum quality controls were discarded. Each sample was aligned to the Human Genome hg38 [18] using bowtie alignment tool [19]. We captured a total of 324 cells across all cell lines. Poor quality cells were removed based on low number of reads as determined using the Fluidigm Singular package (https://www.fluidigm.com/software/). A total of 12 cells were removed. 64 DU145 sensitive cells, 71 DU145 resistant cells, 89 PC3 sensitive cells and 88 PC3 resistant cells were included in all downstream analysis. To identify genes for downstream analysis, we used the Fluidigm Singular package. Genes that were expressed in at less than 10 cells in each cell line were excluded. For the remaining genes, the lowest 15% of expressed genes were excluded. Lastly, genes need to be identified in all four cell line samples to be included in the final gene list. This resulted in 12,862 genes being included for all the subsequence downstream analyses. For gene expression analysis, we followed the Seurat pipeline [20]. In brief, we used this pipeline to conduct dimensional reduction (including PCA and tSNE) on all high-quality single cells using all 12,862 genes. Additionally, we estimated the cell cycle status of each cell using the suggested pipeline for the Seurat package. We used the cell cycle markers included in the Seurat package [21].

### Constructing PANDA regulatory networks

PANDA [15, 22] uses three inputs: a motif prior, a set of known TF-TF interactions, and expression data. To create each cell line specific transcriptional regulatory networks, we ran PANDA with the same TF motif prior data set and TF-TF interaction data, but with gene expression unique to each cell line. To create a motif prior data set, we downloaded the *Homo Sapiens* TF motifs from the Catalog of Inferred Sequencing Binding Preferences CIS-BP [23] for the 240 TFs included in the gene expression data sets. The TF position weight matrices were mapped to the promoter regions of all genes (defined as [− 750:+ 250] around the transcription start site for each gene) using FIMO [24]. To control the TF-TF interaction data set, we estimated the protein-protein interaction network between all 240 TFs using the interaction scores from StringDb v10.5 [25]. All data sources provided in StringDB were included when determining the initial interaction scores. The interaction scores were divided by 1000 and self-interactions were set equal to one. For each network, we constructed a pairwise co-expression levels between each of the target genes (based on Pearson correlation). PANDA then combined this information with the TF motif prior network and TF-TF interaction network to produce each TF regulatory network.

### Specificity score of edges

We identified the enriched edges as calculated in [22]. In brief: for the specificity score (s) of each edge in the regulatory networks, using all four networks, we first calculated the median and interquartile range (IQR) for each edge weight (w) between each TF (t) and gene (g). Next, we compared each individual edge weight (w) to its median and IQR to get the specificity score. An edge was defined as enriched to a network if *s* > *N*. *N* was determined by calculating the specificity scores for the individual genes (*g*) by comparing the median expression of the gene ${w}_g^{(c)}$ in a particular cell line (*c*) to the median and IQR range of the networks constructed from either gene expression data sets. We then varied *N* from 0 to 1. We selected the cut-off of *N* = 0.4 for the single cell sequenced cell lines since at those cut-offs half of all genes are identified as network enriched.

### Node enrichment

To select the TF and gene node enrichment, we followed the method presented in [16, 22]. In brief, each TF was determined to connect with the number of enriched edges (as determined above) in each PANDA network. Using a hypergeometric distribution, we determined which networks targeted a higher number of enriched edges in either network. We calculated the edge weight change by calculating the average edge weight connected to each TF and took the difference between the two PANDA networks. We selected “key” TF and gene nodes that had a *p*-value less than 0.05 in both the network comparison of DU145 and network comparison of PC3. TF and gene nodes must also have both a positive or negative edge weight fold change in the comparison of DU145 and PC3 sensitive and resistant networks to be considered a “key” TF or gene node.

### Gene set enrichment analysis

TF specificity score for each gene was determine using the specificity scores for each edge connected to the specific TF. Then Gene Set Enrichment Analysis (GSEA) was performed as previously described [22, 26]. In our study, we used the list of specific scores for each TF to run a pre-ranked GSEA [26] to test for enrichment of gene ontology terms. Highly significant enriched associations (FDR < 0.05) from these analyses were subjected to hierarchical clustering to group the enriched gene sets into clusters. For each cluster, the frequency that each word appeared in the GO terms assigned to each cluster was determined and used to calculate its statistical enrichment based on its hypergeometric probability. We then scaled the resulting *p*-values by –log_10_ so that the most statistically relevant words would appear the largest. We used the R package wordcloud2 to generate the resulting word clouds for each cluster.

### Connectivity map analysis

We used the Connectivity Map data set (build01) containing genome-wide expression data for 453 treatment and vehicle control pairs, representing 164 distinct small molecules (https://portals.broadinstitute.org/cmap) [27]. Genes were marked as up- or down-regulated based on their average edge weight in the final TF network.

### Drug response

DU145 and/or PC3 docetaxel-resistant cells were plated and cultured in 96-well plates for 16 h (2 × 10^3^ cells/well). Cells were treated with TSA (15 nM; Sigma Aldrich, St. Louis, MO), kaempferol (0.2 μM; Sigma Aldrich), vorinostat (0.15 μM; Tocris, Avonmouth, Bristol) and/or docetaxel (10 nM, Cell Signaling Technology, Danvers, MA) or vehicle (PBS) for 48 h at which time WST-1 solution (Roche Applied Science) was added to the culture medium and incubated for 2.5 h at 37 °C. Absorbance was subsequently determined on a plate reader at a wavelength of 490 nm (Multi-Mode Microplate Reader, SpectraMax M5, Molecular Devices MDS Analytical Technologies).

### Prostate cancer cell growth assay

The cells (3000/well) were seeded in 96-well plates (Corning, New York, NY, USA) in triplicates for 24 h and then the cells were treated with different concentrations of the indicated Trichostatin A (Sigma Chemical Co., St. Louis, MO). The cells were cultured for 3 days. The number of viable cells was measured by Cell Proliferation Reagent WST-1 (Roche Applied Science) as directed by the manufacturer. IC50, the concentration that caused 50% inhibition of cell growth, was calculated with software from AAT Bioquest, Inc. (https://www.aatbio.com/tools/ic50-calculator).

### Mouse experiments

The mouse experiments were approved by the University of Michigan Institutional Animal Care & Use Committee under Protocol 10,366. Docetaxel-resistant PC3 cells in PBS + 50% matrigel GFR (Corning, Corning, New York) were injected subcutaneously into 60 SCID mice (male, 6 to 9 weeks old, mice sourced from the Unit for Laboratory Animal Mice Breeding Colony Managers at University of Michigan (ULAM-BCM) with approval from them for our experiments) at 10^6^ cells per right flank. During experiment, when specified in the approved protocol, mice were anesthetized with isoflurane inhalation at 2.5 mg/kg. Tumor growth was monitored using calipers to measure the length (L) and width (W) and tumor volume was calculated using the formula π/6 x W^2^ x L. On Day 15 post-injection when average tumor volume was 100mm^3^, mice were randomly assigned to one of 4 treatment groups: vehicle 5% DMSO (*n* = 13), 5 mg/kg docetaxel (*n* = 13), 1.5 mg/kg TSA (Sigma, St. Louis, MO), or combination 5 mg/kg docetaxel and 1.5 mg/kg TSA treatment (*n* = 13). Docetaxel was injected intraperitoneally (IP) weekly in the docetaxel alone and combination groups. TSA was injected IP three times per week on Monday, Wednesday and Friday to the TSA alone and TSA-docetaxel Combo group. At the end of experiment, all mice were euthanatized with CO_2_ inhalation and cervical dislocation as secondary method.

### Statistical analysis

For animal experiments, power calculations were performed using Gpower 3.1.9.7 [28]. For a power of 90% and an effect size of 55% with *p* < 0.05, 13 animals per group were required. Statistical comparison among groups were calculated using a mixed-effects analysis for tumor growth over time and a one-way ANOVA followed by Tukey’s Honest Significant Difference for difference in tumor weight. Statistical significance was determined as *p* ≤ 0.05.

## Results

### Identifying network enriched edges

**Fig. 1:**
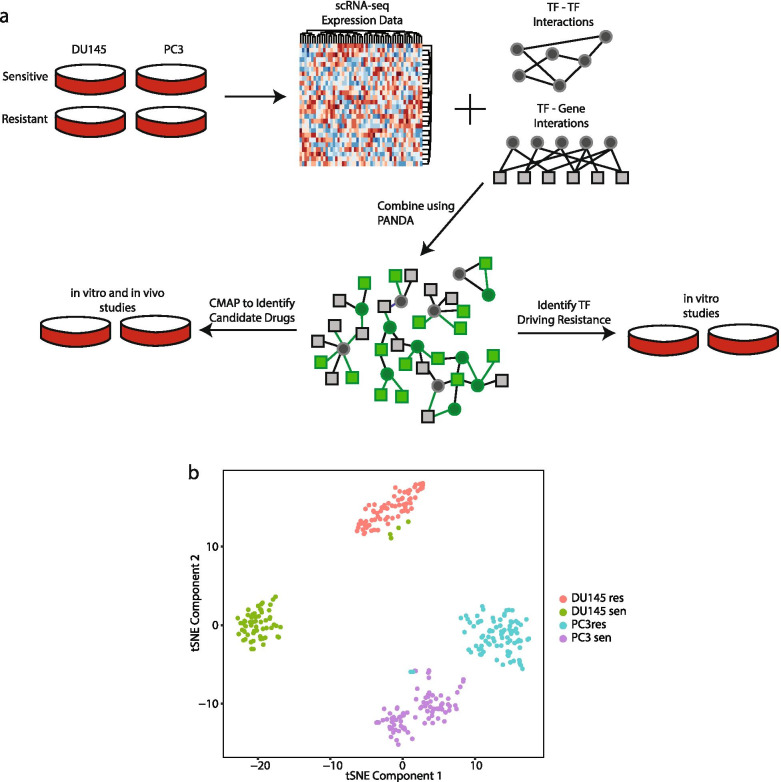
Detailed Workflow of Study. **A** Gene expression data from each scRNA-seq of each cell was combined with physical protein-protein interactions and predicted TF-gene targets to build individual network models. The models from each cell line were compared to identify differences. Shared differences between the DU145 models and PC3 models were combined to create a general model of docetaxel drug resistance. For representation: circles denote TF, squares denote a gene. In the combined network: grey denotes the node or edge is not significantly altered between the sensitive and resistant cells, a green edge or node is statistically significant. **B** tSNE of all single cells analyzed

**Fig. 2:**
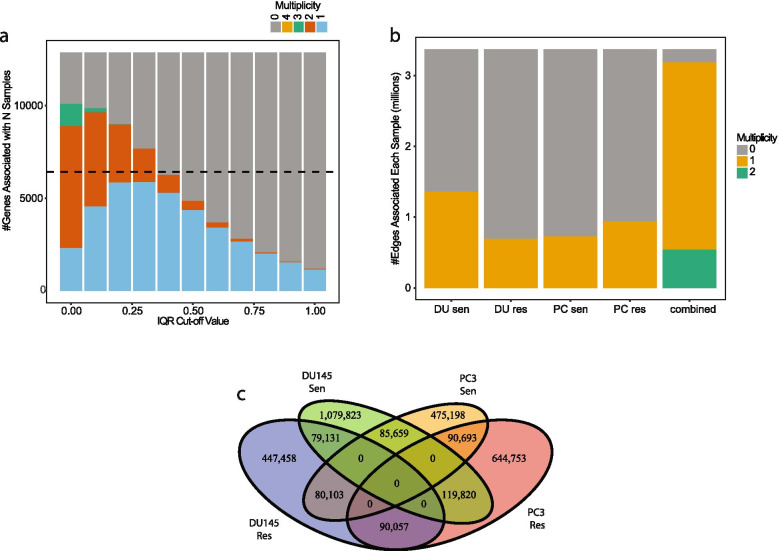
Identification of cell line network enriched edges. **A** Number of genes of a given multiplicity at various cut-offs. **B** Number of network enriched edges in each cell line at a cut-off of 0.4. **C** Venn Diagram of cell line enriched edges

We initially conducted single cell RNA-sequencing on two PCa cell lines, DU145 and PC3, that had been previously created to be resistant to docetaxel treatment [9]. We sequenced both the parental (i.e. docetaxel-sensitive) and docetaxel-resistant variants from both cell lines resulting in a total 312 individual sequenced cells (64 DU145 sensitive cells, 71 DU145 resistant cells, 89 PC3 sensitive cells and 88 PC3 resistant cells). To construct TF activity networks for each of the four established cell lines, we used the PANDA algorithm [15]. PANDA integrates the gene-gene co-expression information from each of the four established cell lines with an initial regulatory network consisting of 240 TF as well as known TF-TF interactions and TF-Gene interactions [25]. This resulted in 4 reconstructed TF regulatory networks (Fig. 1A). We visualized the heterogeneity of all 312 cells and observed that the majority of cells clustered based primarily on their cell line identity (Fig. 1B and Supplementary Fig. S1). We did see a change in the cell cycle phase between the sensitive and resistant cell lines (Supplementary Table S1). We observed an over two-fold decrease in S and G2M phase cells in the resistant compared to sensitive cells from both lines. However, the G1 phase cells increased by over two and half fold in the resistant cells compared to the sensitive cells (Supplementary Table S1). This does suggest a shift in cell cycle stage following docetaxel resistance in PCa cells. To determine which edges and nodes were enriched in the sensitive and resistant DU145 or PC3 networks, we first calculated the interquartile range (IQR) cut-off in which half of the nodes (based on gene expression) would be labeled as network enriched as previously described [22]. We determined this value to be a cut-off of 0.4 (Fig. 2A-B). 3,022,871 edges (89.7%) were enriched in at least one network (Fig. 2B). Of those, only 375,639 edges were enriched for two networks (Fig. 2C). The observation that only 11.1% of the edges overlap between any two networks, indicates that these edges primarily illustrate cell line enriched regulatory interactions across each condition.

### Identifying cell line specific nodes

**Fig. 3:**
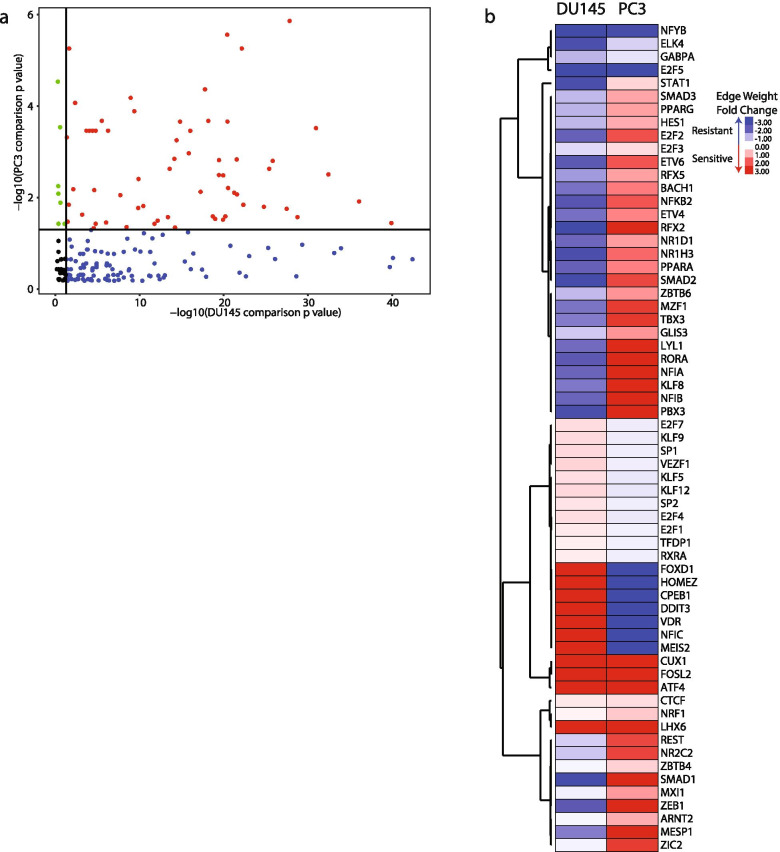
TF nodes altered in both cell line models. **A ***p* values of each TF from network comparison of sensitive and resistant cells lines. Black – not statistically significant, blue – significant only in DU145 network comparison, green – significant only in PC3 network comparison, red – significant in both cell line network comparisons. **B **Heatmap of TFs that were significant in both cell line network comparisons

To determine which TF and gene nodes differed between the sensitive and resistant DU145 or PC3 networks, we compared the ‘in-degree’ of each node as defined as the sum of all edge weights connected to a particular node for each network. We calculated the probability for node statistical significance between the two DU145 or PC3 networks by comparing the number of enriched edges targeted by each node in either network [16]. Of the 240 included TFs, 63 had a *p*-value < 0.05 in both cell line network comparisons (Fig. 3A and Supplementary Table S2). Additionally, we calculated the edge weight fold change based on the ‘in degree’ value for each node. For the 63 TFs, not all had the same fold change direction change for both comparisons. Only 10 of the TFs had an in-degree in the same direction (Fig. 3B).

**Fig. 4:**
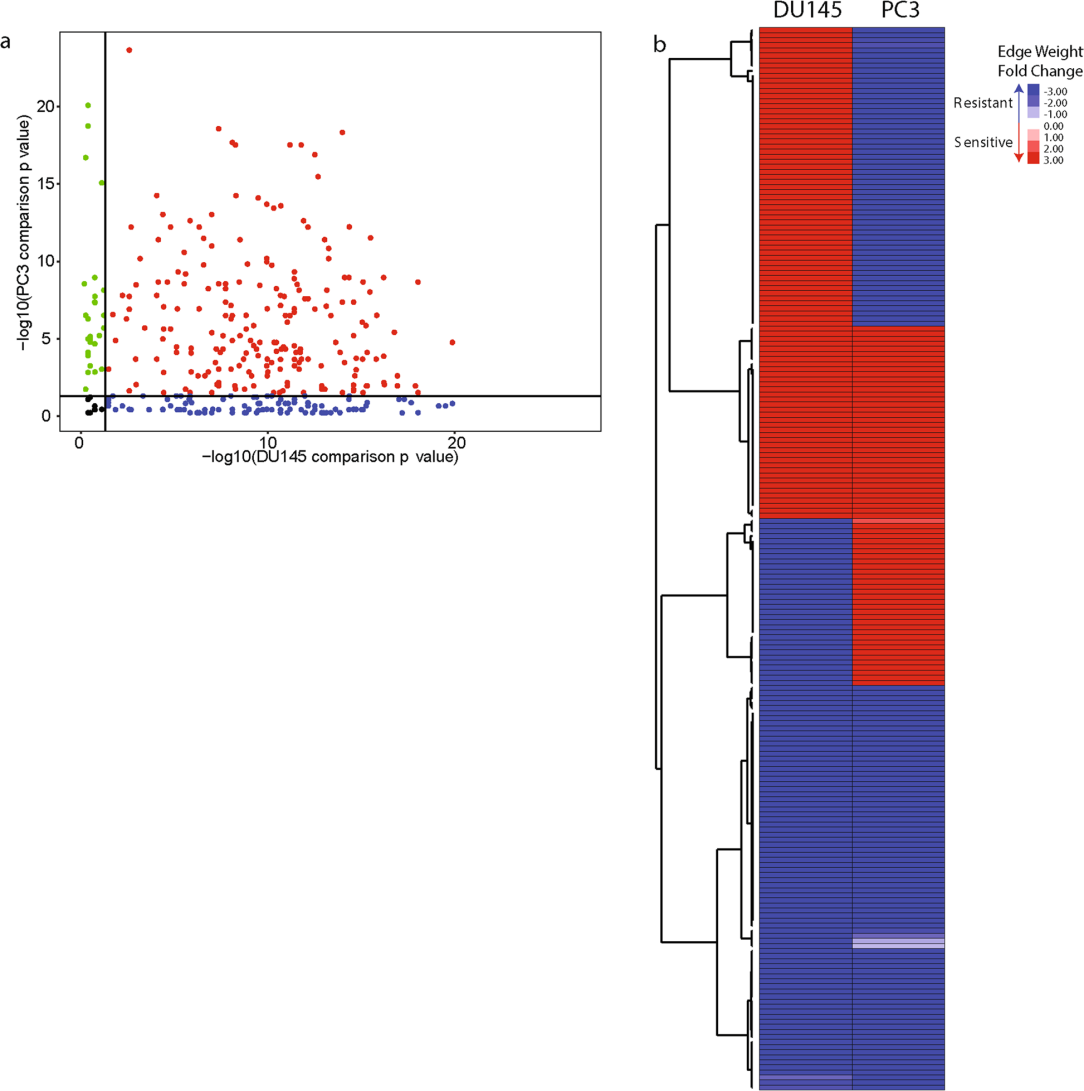
Identification of gene nodes altered in both cell line network models. **A** *p* values of each gene from network comparison of sensitive and resistant cells lines. Black – not statistically significant, blue – significant only in DU145 network comparison, green – significant only in PC3 network comparison, red – significant in both cell line network comparisons. **B** Heatmap of genes that were significant in both cell line network comparisons

We also determined which gene nodes were altered in the TF regulatory networks using the ‘in degree’ values calculated for each gene node. Of the 12,862 genes included in the original gene expression data sets, 210 gene nodes had a *p*-value < 0.05 in both cell line network comparisons (Fig. 4A and Supplementary Table S3). 118 gene nodes had a fold change direction that was the same between the DU145 and PC3 network comparisons (Fig. 4B).

### Regulation of functional pathways

**Fig. 5:**
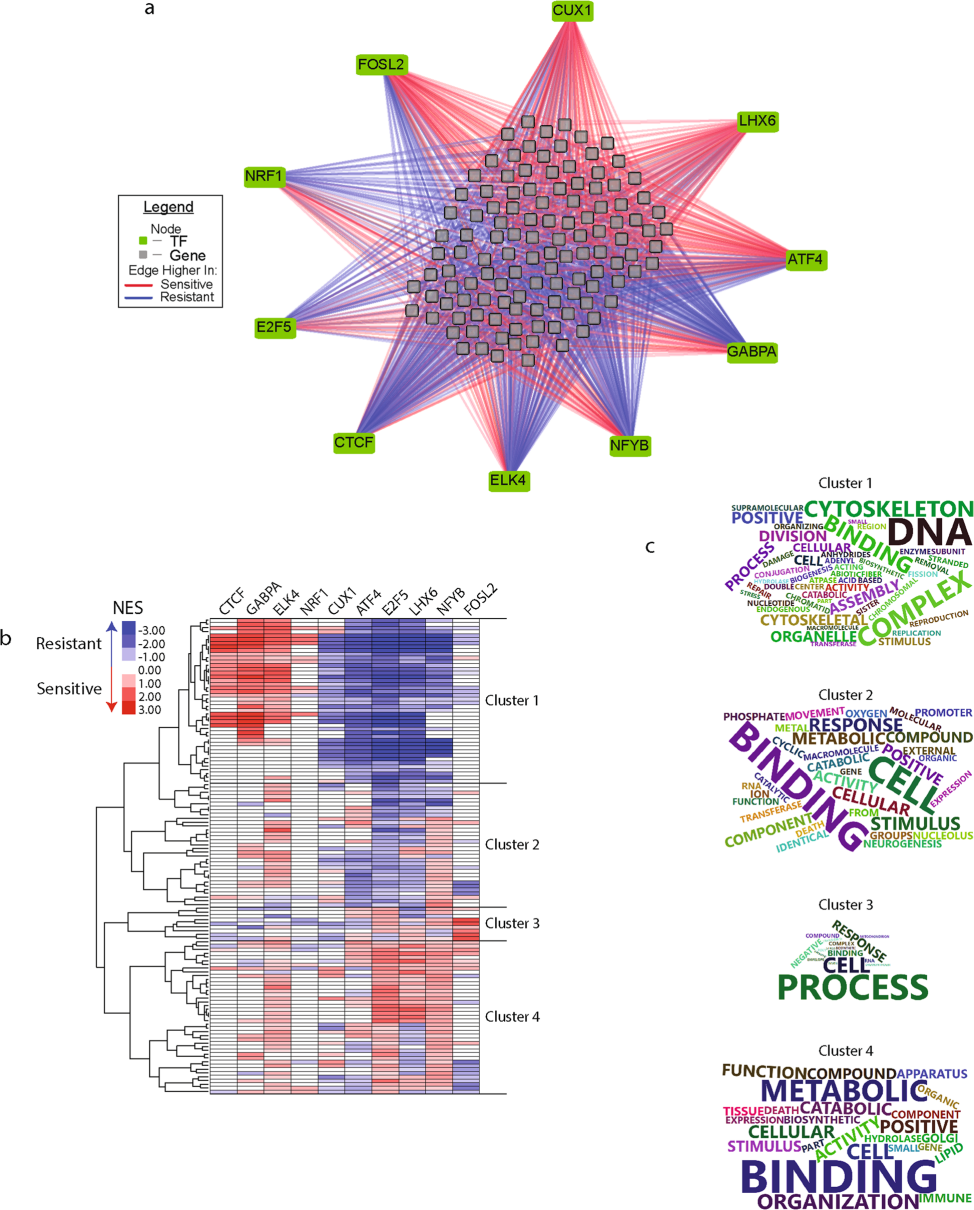
Combined network drive gene pathway changes. **A** Combined network from both cell line comparisons. Edges included were identified in Fig. 2 and connected TF node identified in Fig. 3 and a gene node identified in Fig. 4. **B** Heatmap of gene set enrichment analysis of the sub-network connected to indicated TF. **C** Word cloud of gene ontology names identified in each cluster from (**B**)

To ensure the final network was not representative of just one PCa cell line and to provide robust results, we constructed a final combined TF activity network. This network only includes the common edges and nodes identified in Figs. 2, 3 and 4. Thus, the final TF activity network represents a generalized prostate cancer response to docetaxel treatment (Fig. 5A). In this visualization, the 10 TFs were connected to the 118 gene nodes by lines colored based on whether they exhibit higher average edge weight in the sensitive (red) or resistant (blue) regulatory networks. To explore the functional pathways altered in the regulatory network, we ran gene set enrichment analysis (GSEA) on the TF specific targeted genes to identify four clusters of groups of GO terms (Fig. 5B and Supplementary Table S4). We used word clouds to summarize the GO terms for each cluster to provide a snapshot of the functions (Fig. 5C). These clusters often include sets of highly related functions. Cluster 1 contains GO terms related to the cytoskeleton, chromatin and cellular division suggesting these terms regulate cellular proliferation. In cluster 2, we observe GO terms related to metal ions, signaling molecules and binding suggesting these GO terms regulate cellular signaling. Cluster 3 is the smallest of all the clusters and contains words such as cellular process, envelope, and RNA. This is suggesting this cluster regulates cellular response to signaling but not through any specific mechanism like in cluster 2. Cluster 4 contains words such as metabolic and catabolic suggesting this cluster contains GO terms relating to cellular metabolism Additionally, cluster 1 is enriched by different TFs in the resistant or sensitive cell lines. This may suggest the importance of these pathways regardless of cellular drug sensitivity. In cluster 4, the majority of enrichment was higher in the sensitive cell lines when targeted by the TFs. These data suggest the functional pathway changes that occur during docetaxel resistance due to changes in TF activity.

### Drug combination therapy for drug resistant PCa

**Fig. 6:**
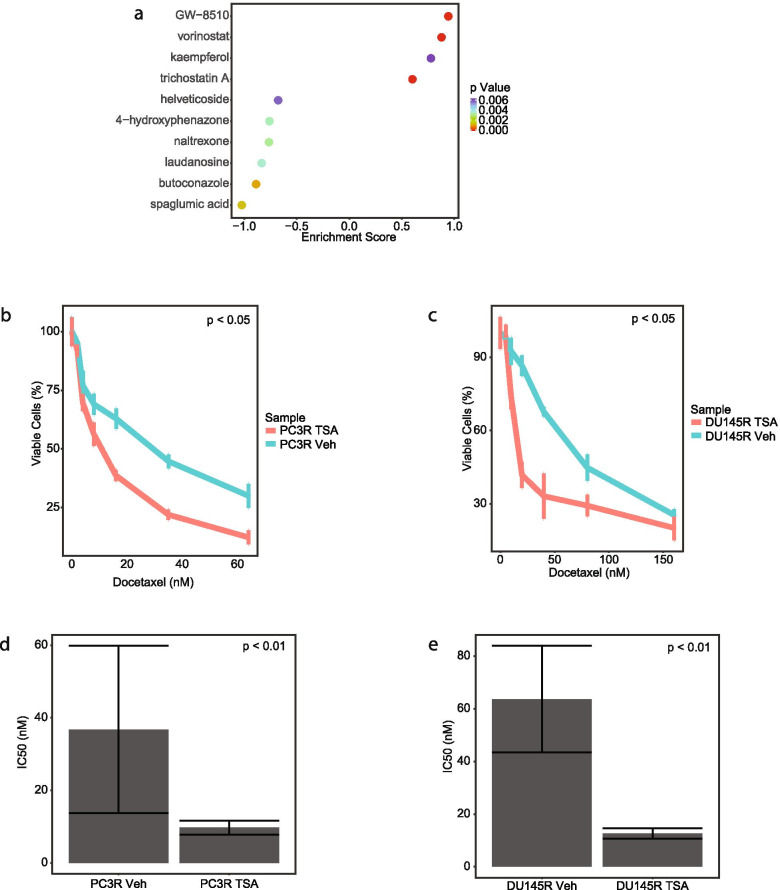
Trichostatin A decreases the resistance to docetaxel. **A** Drugs significantly associated with combined network based on CMAP analysis. **B** Cell viability of PC3 resistant cell line after treatment with docetaxel and trichostatin A. **C** Cell viability of DU145 resistant cell line after treatment with docetaxel and trichostatin A. **D** IC50 of docetaxel of PC3 resistant cell line after treatment with vehicle or trichostatin a. **E** IC50 of docetaxel of DU145 resistant cell line after treatment with vehicle or trichostatin a

**Fig. 7:**
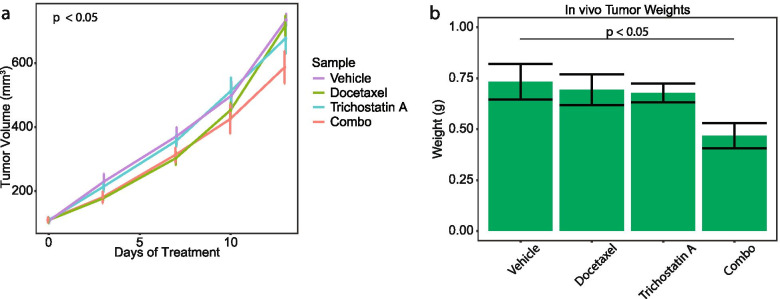
Combination of Trichostatin A and Docetaxel Reduces Tumor Growth in Vivo. **A** Tumor growth of PC3 resistant cell line in mouse model after indicated treatment. **B** Tumor Weight of PC3 resistant cell line in mouse model after indicated treatment

To identify potential alternative clinical therapeutic opportunities in our PCa TF network, we investigated drugs that would potentially disrupt the combined TF regulatory network. To perform this, we labeled the 118 gene nodes as sensitive or resistant nodes based on their edge weight fold change (Fig. 2B). Using this list, we used Connectivity Map (CMAP) analysis to predict drugs that would up-regulate the sensitive genes nodes and down-regulate the resistant gene nodes. The drugs with positive enrichment would be those with the highest potential to reverse docetaxel resistance in PCa. We examined the top hits from CMAP using the final combined regulatory network and identified four potential drugs: vorinostat, GW-8510, kaempferol, and trichostatin A (Fig. 6A and Supplementary Table S5). To investigate these drugs, we tested their ability to overcome docetaxel resistance in the two PCa cell lines. However, we could not evaluate GW-8510 as we were not able to procure it. After determining the IC50 dosage for the remaining three drugs (Supplementary Fig. S2), we found that neither kaempferol nor vorinostat had a significant impact on docetaxel resistance in the PCa cells (Supplementary Fig. S3). However, the combination of trichostatin A and docetaxel statistically significantly decreased the cellular proliferation of both cell lines compared to the vehicle (Fig. 6B-C). Additionally, the IC50 of docetaxel decreased significantly when the same cells were treated with trichostatin A indicating that TSA decreased resistance to docetaxel (Fig. 6D-E). We further tested the combination treatment of trichostatin A and docetaxel in a mouse model of PCa. We established subcutaneous tumors of PC3 resistant cells in mice and then treated the mice with vehicle, docetaxel alone, TSA alone, or the combination of docetaxel and TSA for 13 days. While neither drug alone impacted tumor growth, the combination of TSA and docetaxel decreased tumor growth as measured by both tumor volume (Fig. 7A, *p*-value < 0.05) and final tumor weight (Fig. 7B, *p*-value < 0.05) compared to the three other groups. These data suggest trichostatin A is able to reverse docetaxel resistance in PCa cells in both in vitro and in vivo models.

## Discussion

In this study, we conducted a single cell-based TF network analysis of docetaxel resistance in PCa. We modeled the TF regulatory networks in docetaxel sensitive and resistant PC3 and DU145 PCa cell lines. The final network identified 10 TFs that were the main nodes for the regulatory network. This suggests these TFs are critical for the development of docetaxel resistance in PCa. Furthermore, the gene nodes from the network analysis were subjected to CMAP which suggested that TSA could reverse docetaxel resistant. These findings were validated through use of both in vitro and in vivo models that demonstrated TSA reverses docetaxel resistance. In cell lines, the combination of TSA and docetaxel significantly reduced both the number of viable docetaxel resistant PCa cells and the IC50 value for those resistant cells (Fig. 6). In the PCa mouse model, the combination of TSA and docetaxel reduced tumor growth and final tumor weight greater than either drug alone (Fig. 7). Taken together, these finding demonstrate the validity of this novel methodology of applying network analysis to single cell transcriptomic data to analyze mechanisms of therapeutic resistance and highlights a specific drug that can be tested as a candidate to overcome docetaxel resistance in patients.

The combination of both scRNA-seq and network analysis has enabled the investigation into the TF activity underlying PCa docetaxel resistance. TF activity is regulated beyond the expression level through post-translational modifications including acetylation [29], ubiquitination [30], and sumoylation [31] among others. The application of network analysis, allows for investigation of TF activity beyond just TF gene expression. PANDA of bulk mRNA was previously used to identify the TF co-regulation that existed in triple-negative breast cancer [17] as well as potential biomarkers for anti-angiogenesis treatment in ovarian cancer [16]. In our study, PANDA allowed for determining TF activity networks that identified novel targets for PCa resistance. Our analysis also identified different patterns of TF targeted pathway activation in our GSEA analysis and in the word cloud representation. The word cloud representation identified very high-level terms suggesting that the development of docetaxel resistance impacts very basic cell function. For instance, in cluster 1, a group of gene pathways involved in cytoskeleton and cellular division, we observe one set of TFs driving those pathways. However, in the resistant network, a different set of TFs drive these pathways. This suggests a TF shift from CTCF, GABPA and ELK4 to AFT4, E2F5, and LHX6 among other TFs during drug resistance. Additional study would be needed to explore this TF activity shift.

In our analysis, 10 TFs were identified to be of some statistical significance to either the sensitive or resistant networks. Some of these TFs have been implicated in tumor development or drug resistance in previous studies. CUX1, identified in the sensitive network, is a tumor suppressor and it’s lose promotes tumorigenesis [32]. In PCa, the loss of CUX1 reduces the level of cellular senescence in tumor cells [33]. Combined with our data that suggests a loss of CUX1 activity in resistance cells, CUX1 may play a role in docetaxel resistance in PCa. However, there are multiple TFs with higher activity in the resistant networks such as GABPA, NFYB, and NRF1 among others. GABPA is a downstream target of the androgen receptor in PCa and enables the tumor cells to become more aggressive [34]. NFYB drives paclitaxel resistance in breast cancer [35] and oxalipatin resistance in colorectal cancer [36]. NRF family can been observed to drive cisplatin resistance in pancreatic cancer [37, 38]. Together, this provides evidence that these TFs can drive drug resistance or aggressiveness in tumor cells. And in conjunction with our analyses, could play a role in docetaxel resistance in PCa. However, additional research would be needed to confirm this role.

Applying CMAP to our PANDA network analyses enabled identification of candidate drugs, such as TSA.TSA is a reversible histone deacetylase inhibitor [39] that has been previously shown to be a promising new treatment for other cancers. In osteosarcoma cells, TSA induced cancer cell apoptosis through both histone acetylation- and mitochondria-dependent mechanisms [40]. Interestingly, TSA had a greater specificity in affecting cancer cells compared to normal cells than other histone deacetylase inhibitors [41]. This pro-cancer selectivity makes TSA an attractive therapeutic agent in a clinical setting. Furthermore, TSA enhanced the anti-tumor effects of docetaxel in lung cancer [42]. It was demonstrated that that TSA in combination with docetaxel reduced lung cancer cells by promoting apoptosis. While further work is necessary to determine if a similar mechanism was involved with TSA and docetaxel in PCa, our findings suggest that TSA could overcome docetaxel resistance in PCa cells in patients.

Next generation sequencing is currently used in the clinic to aid in determining potential therapies, such as the identification of mutations or gene fusions, in a precision medicine approach [43, 44]. However, these studies do not identify intra-patient heterogeneity, which plays a role in a patient’s therapeutic response [45, 46]. The addition of single cell sequencing allows for the study of heterogeneity in each patient which could improve a precision medicine approach. Using the analysis pipeline presented in this study, it is possible to identify TF drivers and off-target drug treatments that disrupt individual sub-populations of cells in an individual patient. Further research is needed to determine which sub-populations drive patient treatment responses in order to target the proper sub-populations. Ultimately, this will allow for a personalized therapeutic approach for patients that do not respond to or become resistant to conventional therapies.

## Conclusion

Overcoming drug resistance is critical to improving patient survival in PCa. In this study, we identified a TF activity network common to two different PCa cell lines that drives docetaxel resistance in PCa. We also demonstrated a novel combination therapy to overcome this resistance. This study highlights the usage of novel application of single cell RNA-sequencing and subsequent network analyses that can reveal novel insights which have the potential to improve clinical outcomes.

## Supplementary Information

**Additional file 1: Supplementary Fig. 1**. PCA plot of all sequenced single PCa cells. **Supplementary Fig. 2**. Dosage Curves for potential treatment of PCa cells. (A) Dosage curve for 1 trichostatin A. (B) Dosage curve for vorinostat. (C) Dosage curve for kampferol. 2. **Supplementary Fig. 3**. Combination Treatment of PCa Cells with Potential Drugs and Docetaxel. (A) 3 Proliferation of DU145 resistant cells after treatment of docetaxel and kaempferol. (B) Proliferation of PC3 4 resistant cells after treatment of docetaxel and kaempferol. (C) Proliferation of DU145 resistant cells after 5 treatment of docetaxel and vorinostat. (D) Proliferation of PC3 resistant cells after treatment of docetaxel 6 and vorinostat. *: *p* value < 0.005.

**Additional file 2: Supplementary Table 1**. Cell Cycle Counts Across the Sequenced Single Cells. **Supplementary Table 2**. TF node comparison. **Supplementary Table 3.** Gene node comparison. **Supplementary Table 4**. GSEA Normalized Enrichment Scores. **Supplementary Table 5.** Connectivity Map results

## Data Availability

The single cell RNA sequencing dataset is available from the Gene Expression Omnibus under GSE140440 [https://www.ncbi.nlm.nih.gov/geo/query/acc.cgi?acc=GSE140440].

## Abbreviations

PCa: Prostate cancer
TF: Transcription factor
CMAP: Connectivity map
TSA: Trichostatin A
PANDA: Passing Attributes between Networks for Data Assimilation
CRPC: Castration-resistant prostate cancer
scRNA-seq: Single cell RNA-sequencing
GSEA: Gene Set Enrichment Analysis
FDR: False discovery rate
GO: Gene ontology
IQR: Interquartile range
ULAM-BCM: Unit for Laboratory Animal Medicine Breeding Colony Managers
