# Supplementary figures and images for: Transcription factor network analysis based on single cell RNA-seq identifies that Trichostatin-a reverses docetaxel resistance in prostate Cancer
Source: BMC Cancer. 2021 Dec 8;21:1316. doi: 10.1186/s12885-021-09048-0 (PMC8653542; doi:10.1186/s12885-021-09048-0)

Supplementary Figure 1

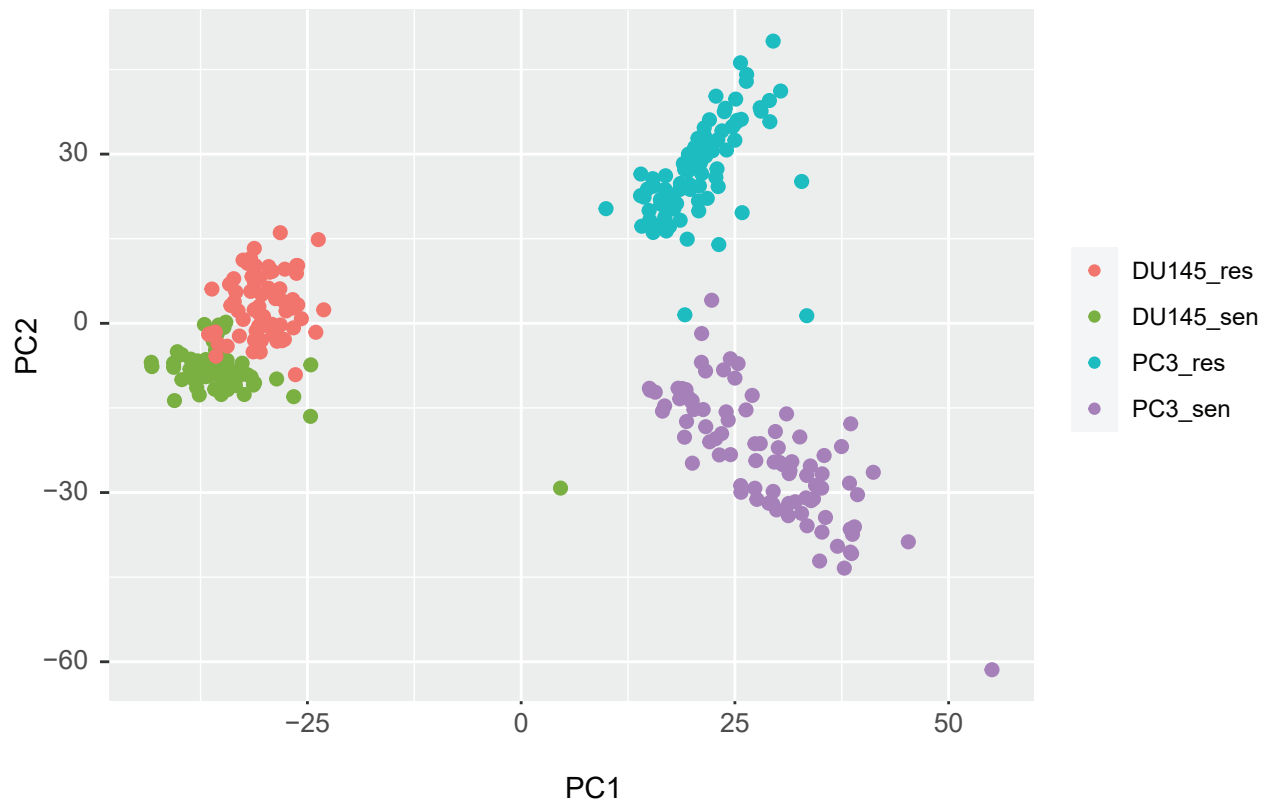

Supplementary Figure 2

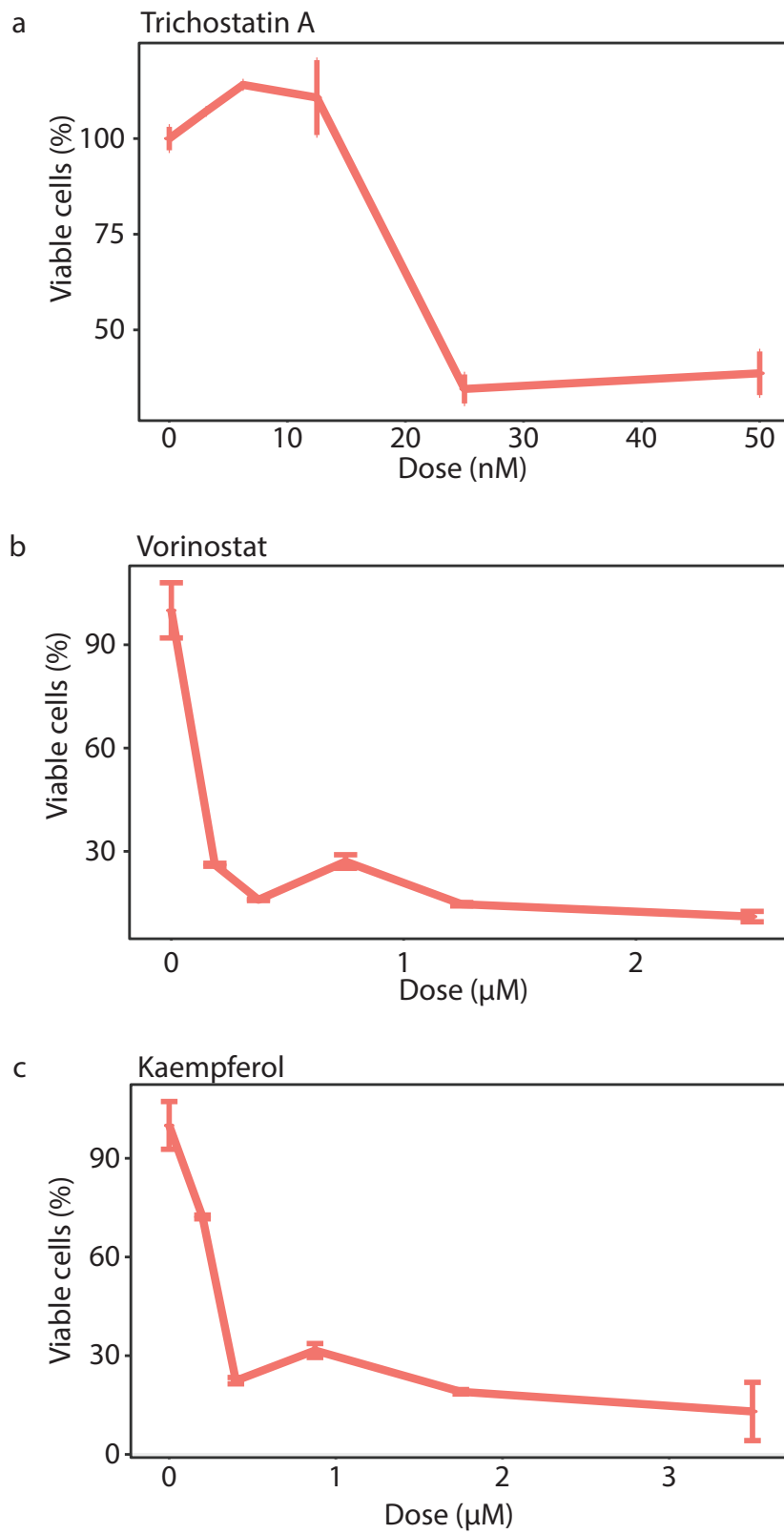

Supplementary Figure 3

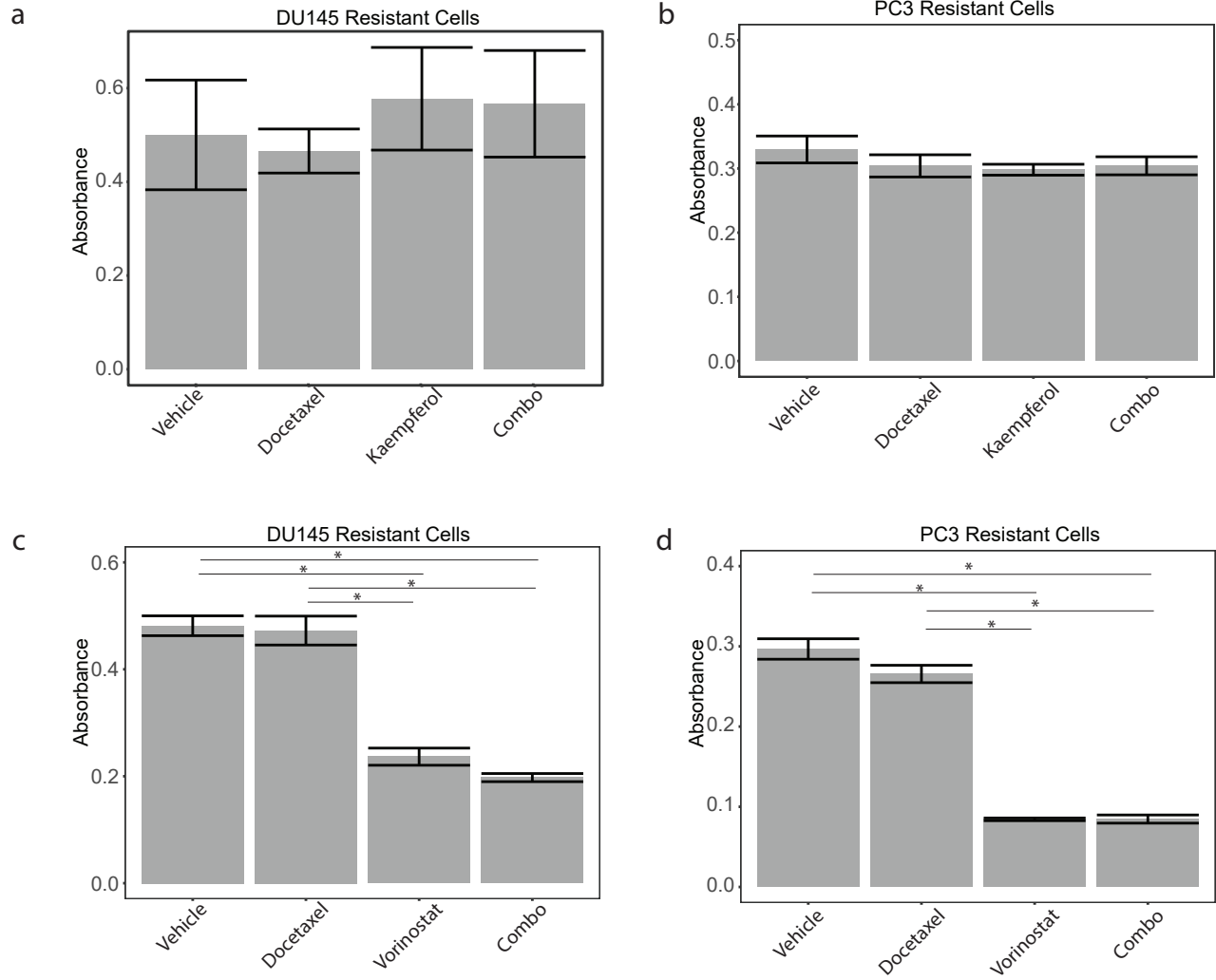

Supplement: Supplementary file 1 — Additional file 1: Supplementary Fig. 1. PCA plot of all sequenced single PCa cells. Supplementary Fig. 2. Dosage Curves for potential treatment of PCa cells. (A) Dosage curve for 1 trichostatin A. (B) Dosage curve for vorinostat. (C) Dosage curve for kampferol. 2. Supplementary Fig. 3. Combination Treatment of PCa Cells with Potential Drugs and Docetaxel. (A) 3 Proliferation of DU145 resistant cells after treatment of docetaxel and kaempferol. (B) Proliferation of PC3 4 resistant cells after treatment of docetaxel and kaempferol. (C) Proliferation of DU145 resistant cells after 5 treatment of docetaxel and vorinostat. (D) Proliferation of PC3 resistant cells after treatment of docetaxel 6 and vorinostat. *: p value < 0.005. [file 12885_2021_9048_MOESM1_ESM.pdf]
